# A novel repetitive head impact exposure measurement tool differentiates player position in National Football League

**DOI:** 10.1038/s41598-019-54874-9

**Published:** 2020-01-28

**Authors:** Clara Karton, T. Blaine Hoshizaki, Michael D. Gilchrist

**Affiliations:** 1 University of Ottawa, Health Sciences, Ottawa, K1N 6N5 Canada; 2 University College Dublin, School of Mechanical and Materials Engineering, Belfield, Dublin 4 Ireland

**Keywords:** Cell death in the nervous system, Trauma, Public health, Brain injuries

## Abstract

American-style football participation poses a high risk of repetitive head impact (RHI) exposure leading to acute and chronic brain injury. The complex nature of symptom expression, human predisposition, and neurological consequences of RHI limits our understanding of what constitutes as an injurious impact affecting the integrity of brain tissue. Video footage of professional football games was reviewed and documentation made of all head contact. Frequency of impact, tissue strain magnitude, and time interval between impacts was used to quantify RHI exposure, specific to player field position. Differences in exposure characteristics were found between eight different positions; where three unique profiles can be observed. Exposure profiles provide interpretation of the relationship between the traumatic event(s) and how tissue injury is manifested and expressed. This study illustrates and captures an objective measurement of RHI on the field, a critical component in guiding public policy and guidelines for managing exposure.

## Introduction

Various levels of impact severity associated with head injury have been studied for over a century where knowledge and reporting reflected current periods’ public concern and environmental context^1^. The scientific community, medical sectors and regulatory authorities have been working for decades to unravel the complexity of head injury in sport, most predominantly the definition, identification, and treatment of concussions. There were obvious challenges associated with the ambiguous nature and presentation of what is regarded as a tissue ‘injury’^2,3^. Inconsistencies in symptom expression and prediction, and human variability in predisposition limit the precision in defining the relationship between the traumatic event(s) and injury. Despite numerous reports describing the implications of the acute and chronic effects of single and repeated sub-concussive and concussive blows to the head^1^, only recently have exposure to repetitive head impacts (RHI) in contact sports become a real concern, and this concern has become relevant to researchers, neurologists, clinicians and athletic professionals alike. The high profile sport, American-style football (ASF), has received increasing attention amongst researchers who believe that this population will help establish a better understanding of the consequences and management of RHI.

Measuring head injury in ASF solely on the basis of immediate signs and symptoms has proven to be insufficient in capturing the spectrum of tissue injury^4,5^. Moreover, as individuals recover from the clinical expression of symptoms, the assumption that neurobiological recovery has also occurred is also likely to be inaccurate^6,7^. A more sophisticated understanding that now prevails considers the time course of neurobiological recovery and the mental health consequences of being exposed to RHI. Oliver and colleagues^8^ report that blood concentrations of axonal injury biomarkers in college football starters remain elevated even 9 weeks post season. The scientific debate regarding head injury in ASF has progressed from how concussion is managed, to how a person’s cumulative history of neurotrauma, inclusive of sub-concussive impacts, should be managed.

Not only are a large percentage of concussions^9,10^ unrecognized or underreported, but a number of modalities have been used to show changes in brain function, connectivity, activation and cognition, following sub-concussive RHI^11–14^. Many deficits associated with the long-term consequences of RHI are not clinically apparent until years after the impacts are experienced^15,16^. This has raised awareness of the link between cumulative RHI and the development and/or initiation of neurological disorders, including chronic traumatic encephalopathy (CTE). CTE has been associated with exposure to RHI, and often presents with cognitive, motor and psychiatric related deficiencies^17–20^. The vulnerabilities associated with duration of exposure to RHI are implicated in a number of reports showing higher plasma tau concentrations, increased cognitive impairments and reduced microstructural integrity of the corpus callosum found in retired NFL players compared to their age matched controls^4,16,18,21,22^.

Professional ASF is associated with one of the highest rates of documented concussion and the development and pathology of neurological disorder within sport^20,23,24^, and currently accounts for the greatest number of post-mortem diagnosed cases of CTE^25,26^. This self-selection biased data set, however, makes it difficult to determine the true incidence and risks associated with RHI leading to CTE development, particularly within the ASF population. However, a recent quantitative risk assessment concluded that CTE poses a public health concern, and suggests that regardless of the limited data on causation, incidence, and/or the dose-response relationship, reducing the exposure to RHI in ASF would consequently result in a reduction in the occurrence of brain disease^27^. It is the societal impact of RHI that will govern how public health is managed and public policy and clinical guide lines are set. This underlines the importance of developing a more effective and consistent way to quantify RHI in ASF and define tissue injury^28^.

Research examining RHI leading to metabolic, physiologic and structural alterations of neuronal cells propose an interaction between exposure characteristics that create risk for a person’s brain health. Biomarker assays and various imaging techniques are sufficiently sensitive to detect changes in athletes who experience both concussive and sub-concussive head impacts^8,11,12,29^. The degree of alteration associates with both the intensity of impact and number of impacts received^7,13,14,30–32^. Moreover, taking time for brain tissue to recover from either frequent or intense impacts has been implicated in recurrent concussion and brain health^8,33–35^. Frequency, magnitude, and interval characterize RHI and are important and relevant to long-term psychiatric sequelae. Head contact is integral to ASF, resulting in hundreds of RHI throughout a season^36–39^. Biomechanical assessment of RHI that incorporate multiple event characteristics have predominantly been derived from self-reported histories and head impact sensors to estimate exposure^5,15^. Innovations designed to measure the dynamic response of the head resulting from impacts report RHI estimates for youth, high school and collegiate level ASF, however exposure at the professional level has not been documented^38–42^. Wearable head impact sensors allow for large amounts of data collection in real-time and can provide information in terms of impact count/frequency, however pose valid concerns regarding their accuracy for measuring dynamic magnitudes and duration, particularly evident for rotational head motion^43^. These technologies are limited in predicting the brain’s response^44,45^, consequently present challenges for interpreting exposure results. Physical head impact event reconstruction and finite element analysis provide useful information to investigate the link between head motion and brain tissue strain^46,47^.

The number, type and magnitude of impacts received will naturally vary based on a player’s positional responsibilities^37,38^. Field positions and plays create environments that dictate a number of characteristics of the traumatic events unique to play position and are implicated in the documented rates and risks of concussion and brain disease^23–25^. This variability in position provides an opportunity to examine how the characteristics of RHI may contextually advance the understanding of how tissue injury is manifested and expressed.

The purpose of the current study was to employ a novel method for quantifying RHI using a biomechanical measurement method that incorporates frequency of impact, tissue strain magnitude and time interval between impacts. This method was used to objectively measure RHI exposure specific to player field positions in professional ASF.

## Results

Kruskal-Wallis H tests were conducted to examine any statistically significant effects of player field position on impact frequency, strain magnitude, time interval and cumulative RHI as brain strain exposure per time (BSE/T). Additionally, a contingency table analysis was used to determine differences in frequency distributions of strain magnitude experienced by each position. Eight ASF field positions were included: Quarterback (QB), Running Back (RB), Wide Receiver (WR), Tight End (TE), Offensive Line (OL), Defensive Line (DL), Linebacker (LB), and Defensive Back (DB).

### Characteristics of RHI exposure

#### Frequency of head impacts

A total of 3439 (2941 confirmed; 498 suspected) head impacts were documented for eight player positions during 32 regular season games (Table 1). Frequencies are presented per position based on total frequency, average per game, and estimated frequency for a 16-regular game season in professional ASF (excludes practices). The Kruskal-Wallis H test showed strong evidence of a statistically significant difference in impact frequency between the mean ranks of player position, χ^2^ (7) = 187.21, p = 0.000: QB mean rank = 43.50; RB mean rank = 150.09; WR mean rank = 56.53; TE mean rank = 170.64; OL mean rank = 200.89; DL mean rank = 213.44; LB mean rank = 134.02; DB mean rank = 58.89. Dunn’s pairwise showed specifically that differences were between QB, WR, DB, and the remaining five positions (Table 1). Additionally, significant differences were found between LB and OL (p = 0.008); LB and DL (p = 0.000); and RB and DL (p = 0.017). Independent of position, the average number of impacts a player received during game play over a 16-game season was estimated at approximately 184 impacts. Impact frequencies are estimated for an average professional football career (ave. yrs.) and for the greatest number of games played (># games) documented in NFL player history (Table 1; Pro-Football-Reference.com, 2014; 2018). Suspected and multiple (pile-up) impacts are presented for the eight positions (Table 1). Suspected impacts accounted for approx. 15% of the total impacts recorded; RB with the greatest number recorded, followed by LB and DL. RB entered a pile-up twice as many times as the closest second (DL).This results in a conservative estimate of overall frequencies.

**Table 1:** Head impact frequency counts for ASF positions documented from 32 regular season games played from 2009–2015.

Player Position	Confirmed Impacts			Suspected Impacts	Pile-up	NFL Career			
	Total freq. #	Per Game (ave. ± SD)	Per Season (16 games)			ave. yrs.	est. freq. #	># games	est. freq. #
QB	73	2.3 ± 2.0	36.5	23	7	3.08	112	302	694.6
RB^a***^	468	14.6 ± 5.4	234	186	106	2.42	566.3	226	3299.6
WR	106	3.3 ± 2.9	53	33	7	2.21	117.1	303	999.9
TE^a***^	459	14.3 ± 6.1	229.5	34	10	2.67	612.8	270	3861.0
OL^a***b*^	637	19.9 ± 8.3	318.5	31	15	3.67	1168.9	296	5890.4
DL^a***b***c*^	706	22.1 ± 8.5	353	71	53	3	1059	282	6232.2
LB^a***^	377	11.8 ± 4.6	188.5	82	22	3	566	278	3280.4
DB	115	3.6 ± 2.5	57.5	38	10	3.17	182	295	1062.0

*significant at p < 0.05; **significant at p < 0.005; ***significant at p < 0.001.

^a^significant difference to QB, WR & DB, ^b^significant difference to LB, ^c^significant difference to RB.

**Figure 1:**
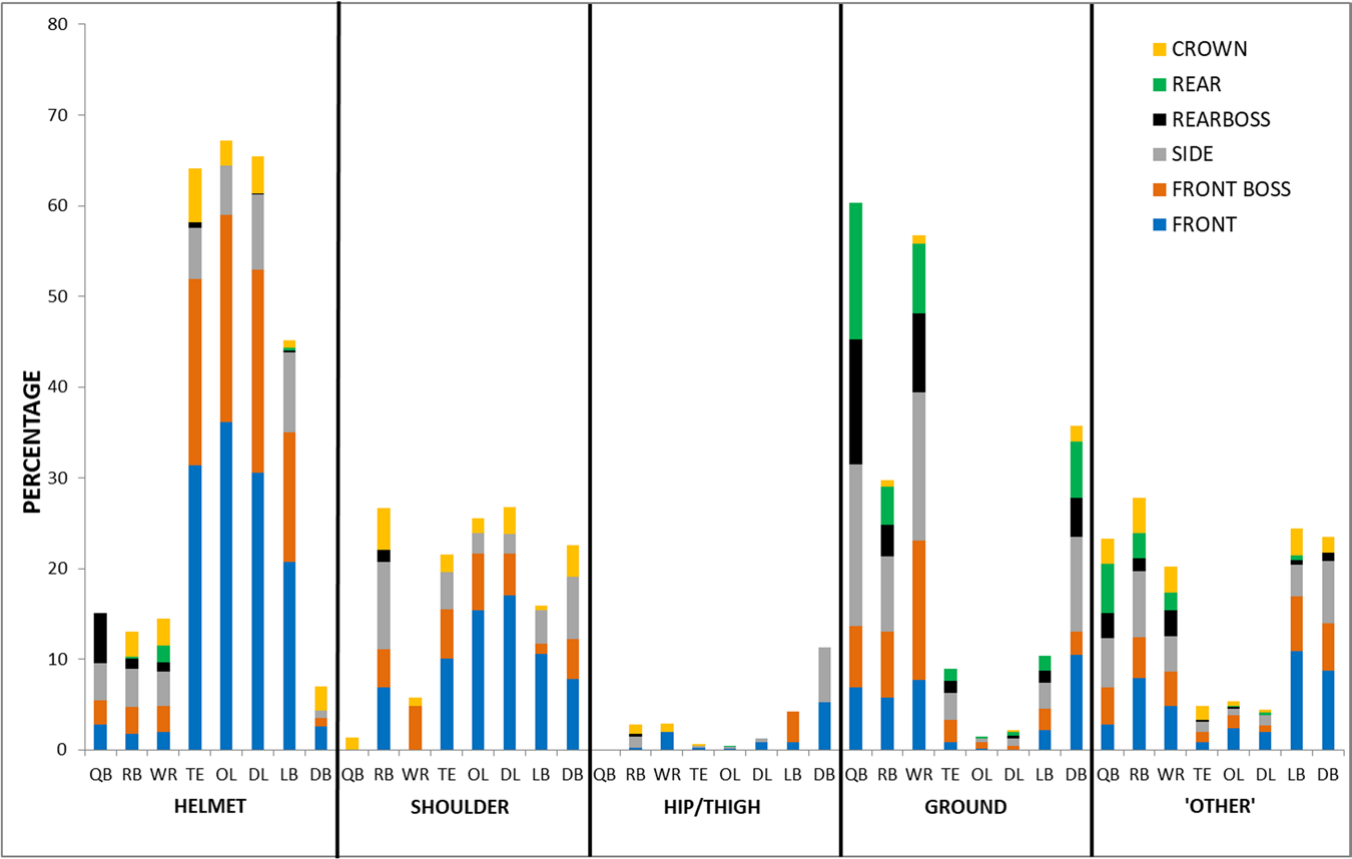
The distribution of head impact event type and head location for eight player positions in professional ASF captured from 32 regular season games. Distributions are presented as a percentage from the total number of impacts specific to each position.

With player position collapsed, helmet represent 49% of the total documented impacts. Shoulder impacts were the second most common event type with 23% of the total. The remaining two event categories accounted for the fewest documented impacts (ground = 13%, hip/thigh = 2%). ‘Other’ events described 13% of impacts (back = 3.4%, chest = 1.7%, stomach = 1%, arm = 2.5%, hand = 2.5%, leg = 1.2%, knee = 0.6%, field goal post/wall = 0.1%). Most often LB, TE, OL and DL experience helmet impacts, and these were predominantly to the front location (Fig. 1). Impacts with the ground are more frequent among the QB, WR and DB positions, accompanied by a noticeable shift to include the side and rear head locations. An even distribution between shoulder and ground event types can be observed for the RB. This position also experienced recurrent head impacts to their opponents’ torso represented in the ‘other’ category (Fig. 1). Side location was greatest for the RB player position.

#### Magnitude of brain tissue maximum principal strain (MPS)

The maximum principal strain (MPS) resulting from exemplar impacts for eight ASF field positions ranged from 4.6–50.6%. The mean MPS resulting from exemplar reconstructions of each event type individually and collapsed for each ASF position is presented in Table 2. MPS resulting from all exemplar impacts was further categorized into five levels of magnitude: very low = <8%, low = 8–16.9%, moderate = 17–25.9%, high = 26–34.9%, very high = 35%+.

**Table 2:** Maximum principal strains (%) resulting from exemplar physical reconstructions of common head impacts specific to ASF field position.

Field Position	Event Type				Collapsed
	Helmet	Shoulder	Hip	Ground	
	Mean MPS with 95% CI				
QB	24.1, [15.9, 32.3]	11.9	—	27.4, [19.5, 35.3]	25.4, [19.7, 31.1]
RB	25.3, [18.7, 31.9]	16.3, [13.4, 19.2]	10.4, [4.5, 16.3]	18.0, [12.0, 24.0]	17.1, [11.8, 22.5]
WR	22.5, [0.139, 31.1]	14.1, [13.5, 14.7]	8.7, [4.9, 12.5]	25.2, [18.1, 32.3]	21.8, [16.9, 26.7]
TE	19.5, [14.4, 24.6]	17.0, [13.3, 20.7]	11.5, [3.8, 19.2]	22.7, [14.8, 30.6]	19.1, [15.8, 22.4]
OL	12.7, [8.3, 17.1]	14.1, [9.3, 18.9]	7.5, [3.7, 11.3]	20.8, [12.1, 29.5]	14.3, [11.1, 17.5]
DL	19.7, [14.0, 25.4]	17.6, [13.3, 21.9]	9.6, [0.8, 18.4]	12.5, [8.3, 16.7]	16.3, [13.4, 19.2]
LB	18.4, [14.3, 22.5]	18.3, [10.1, 26.5]	21.3, [7.6, 35.0]	17.3, [10.5, 24.1]	18.3, [14.9, 21.7]
DB	0.242, [17.8, 30.6]	18.0, [12.2, 23.8]	14.9, [0.5, 29.3]	23.5, [16.7, 30.3]	21.1, [17.1, 25.1]

Results are presented as sample means. Values in square brackets indicate 95% Confidence Intervals.

Statistically significant rank mean differences in exemplar impact magnitudes with event type collapsed, were found between ASF player position (χ^2^ (7) = 15.68, p = 0.028): QB mean rank = 172.82; RB mean rank = 123.57; WR mean rank = 139.88; TE mean rank = 128.64; OL mean rank = 92.67; DL mean rank = 109.23; LB mean rank = 122.30; DB mean rank = 138.17. Post hoc identified one significant difference between QB and OL (p = 0.016).

**Figure 2:**
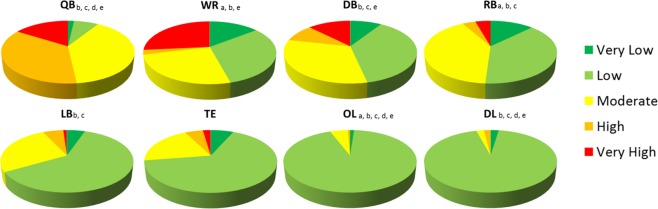
Distribution of five MPS levels of magnitude for eight player positions. Distributions are based on physical reconstruction and finite element analysis from the total impact frequencies documented as confirmed head impacts of 4 common event types from 32 games. All are significant at p < 0.001. a = significant difference in ‘very low’ (<8% MPS), b = significant difference in ‘low’ (8–16.9% MPS), c = significant difference ‘moderate’ (17–25.9% MPS), d = significant difference in ‘high’ (26–34.9% MPS), e = significant difference in ‘very high’ (35 + % MPS).

Significant association between distribution within magnitude level and field position was identified using Pearson chi-square statistic in a contingency table analysis (Fig. 2; χ^2^ (28) = 1066.27, p = 0.000) (Fig. 2). RB (*z* = 8.27, p = 0.000) and WR (*z* = 4.69, p = 0.000) experienced a higher percentage of ‘very low’ level impacts; OL showed a statistically lower distribution of ‘very low’ impacts (*z* = −4.30, p = 0.000) in comparison to the group mean. A significantly lower distribution of ‘low’ level impacts were found for QB (*z* = −11.46, p = 0.000), RB (*z* = −15.92, p = 0.000), WR (*z* = −8.94, p = 0.000), LB (*z* = −5.03, p = 0.000), and DB (*z* = −7.87, p = 0.000), and a higher percentage for OL (*z* = 12.29, p = 0.000) and DL (*z* = 13.56, p = 0.000). QB (*z* = 5.13, p = 0.000), RB (*z* = 13.21, p = 0.000), LB (*z* = 5.65, p = 0.000) and DB (*z* = 4.48, p = 0.000) were above group mean percentage for ‘moderate’ magnitude level impacts. ‘High’ level impacts accounted for a significantly larger distribution for QB (*z* = 11.87, p = 0.000), and the percentage of ‘very high’ magnitude impacts were statistically higher for QB (*z* = 6.20, p = 0.000), WR (*z* = 13.52, p = 0.000), and DB (*z* = 5.73, p = 0.000) when compared to the group means. The percentage of impacts for OL and DL were statistically lower for ‘moderate’ (*z* = −7.78, p = 0.000; *z* = −10.85, p = 0.000, respectively), ‘high’ (*z* = −4.96, p = 0.000; *z* = −4.44, p = 0.000, respectively), and ‘very high’ (*z* = −4.70, p = 0.000; *z* = −5.07, p = 0.000, respectively) magnitudes.

#### Time interval between head impacts

Time between impacts is presented as averages per game, both as an overall time and distributed by MPS magnitude level (Table 3). Strong evidence of statistically significant differences in time interval between the mean ranks of player field positions was identified by the Kruskal-Wallis H test (χ^2^ (7) = 94.49, p = 0.000); QB mean rank = 178.08; RB mean rank = 120.19; WR mean rank = 155.42; TE mean rank = 93.66; OL mean rank = 62.09; DL mean rank = 46.19; LB mean rank = 118.11; DB mean rank = 153.55. Post hoc analysis showed time interval (collapsed magnitude) for OL and DL was different compared to all remaining positions excluding TE (Table 3). The TE position showed statistical difference to QB (p = 0.001), WR (p = 0.013), and DB (p = 0.019).

**Table 3:** Average time interval (min) between impacts and head impact frequency (#) per game, distributed by MPS magnitude level.

		Magnitude Collapsed		Magnitude Category (MPS)									
				<8.0%		8.0–16.9%		17.0–25.9%		26.0–34.9%		35.0%+	
			*n*		*n*		*n*		*n*		*n*		*n*
QB^a***b***c**^	#	1.75 ± 1.72		0.03 ± 0.18		0.13 ± 0.34		0.69 ± 0.86		0.63 ± 0.91	5	0.28 ± 0.52	1
	min	30.3 ± 14.65	13	–		—		53.88 ± 28.50	6	28.53 ± 19.03		30.33	
WR^a***b***c*^	#	2.59 ± 2.28		0.38 ± 0.71	1	0.81 ± 0.93		0.66 ± 0.94		0.06 ± 0.25		0.69 ± 0.86	4
	min	25.68 ± 24.18	19	47.61		29.44 ± 13.47	7	35.39 ± 25.56	5	—		39.42 ± 23.34	
DB^a***b***c*^	#	2.75 ± 2.33		0.25 ± 0.67	2	1.03 ± 1.20		0.88 ± 1.10	7	0.25 ± 0.44		0.34 ± 0.70	2
	min	24.69 ± 21.53	19	16.29 ± 23.02		37.78 ± 30.51	8	48.61 ± 38.67		—		66.68 ± 0.13	
LB^a***b*^	#	8.91 ± 3.49		0.47 ± 0.72	4	5.47 ± 2.76		2.34 ± 1.52		0.53 ± 0.76	5	0.09 ± 0.30	
	min	13.16 ± 7.19	31	64.69 ± 34.08		21.21 ± 22.42	31	29.82 ± 18.31	22	43.94 ± 48.21		–	
RB^a***b**^	#	10.59 ± 4.05		1.34 ± 1.33	10	4.06 ± 2.54		4.13 ± 2.17		0.59 ± 0.71	4	0.47 ± 0.67	3
	min	13.25 ± 6.90	32	37.47 ± 19.76		32.05 ± 27.82	31	25.78 ± 14.42	27	48.90 ± 34.04		61.67 ± 37.96	
TE	#	13.66 ± 6.31		0.22 ± 0.42		10.41 ± 5.47		2.00 ± 1.68		0.72 ± 0.92	6	0.31 ± 0.59	2
	min	10.37 ± 5.20	32	–		14.77 ± 12.41	31	36.37 ± 25.68	17	32.23 ± 11.43		33.74 ± 46.53	
OL	#	18.84 ± 8.33		0.22 ± 0.49	1	17.53 ± 8.06		0.97 ± 1.75		0.13 ± 0.34		—	
	min	7.81 ± 4.61	32	19.31		8.30 ± 5.91	31	24.28 ± 14.76	7	—		—	
DL	#	21.19 ± 7.83		0.50 ± 0.67	3	19.81 ± 7.38		0.47 ± 0.76		0.41 ± 0.71	4	—	
	min	6.29 ± 2.13	32	14.25 ± 9.22		6.73 ± 2.59	32	54.99 ± 29.29	3	80.69 ± 24.80		—	

Interval per magnitude level was calculated only for games in which >2 head impacts of the respective magnitude was experienced during the same game. The number of games per position for interval calculations is indicated with *n* values. Averages are presented (±SD). Significance is presented for differences in interval between positions.

*Significant at p < 0.05; **Significant at p < 0.005; ***Significant at p < 0.001.

^a^significant difference to DL, ^b^significant difference to OL, ^c^significant difference to TE.

### Cumulative RHI exposure

**Figure 3:**
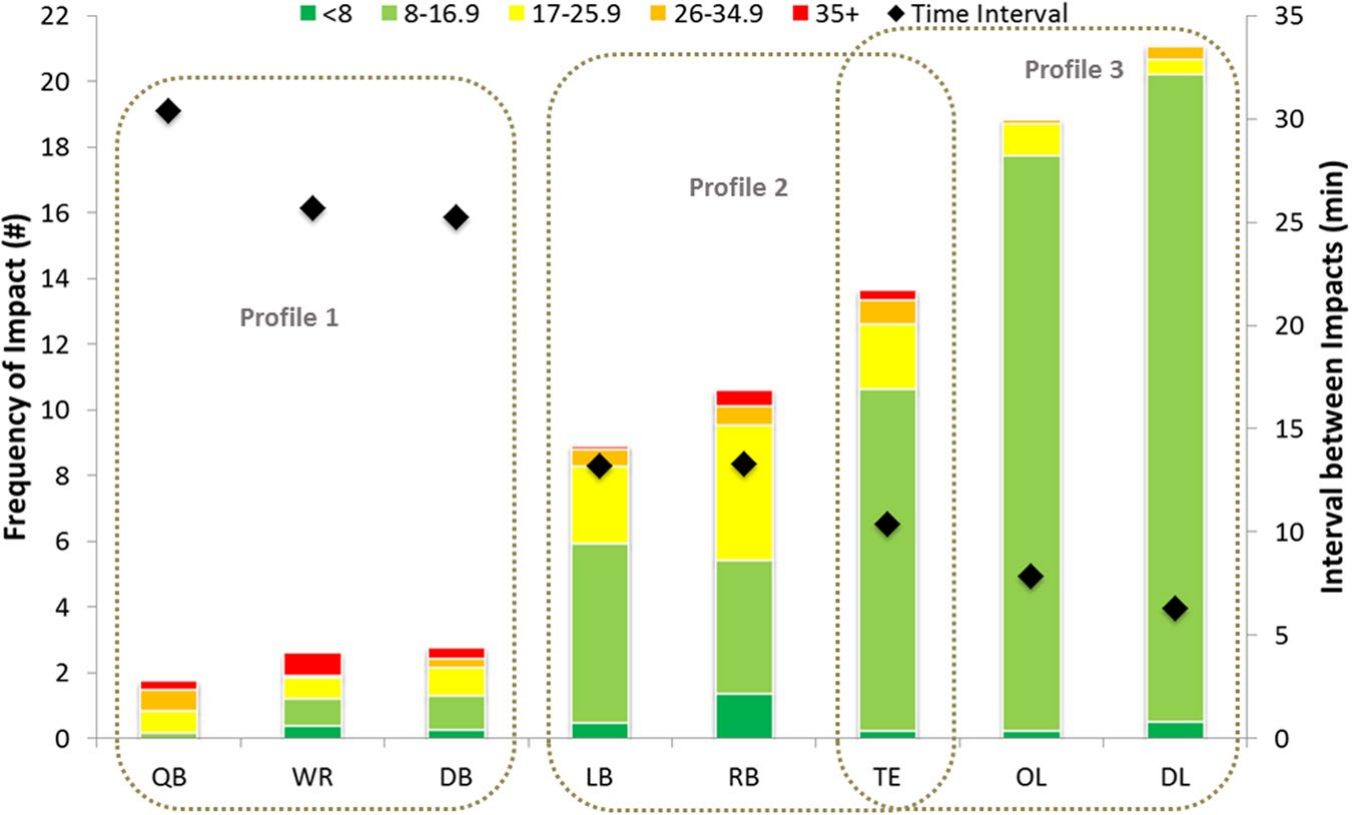
Position specific BSE/T profiles presented based on per game averages. Average head impact frequency distributed by MPS (%) magnitude. Interval is presented as an average time (measured in minutes) between impact frequency total (magnitude collapsed).

Statistical differences in the mean ranks of BSE/T were found between the ASF field positions (χ^2^ (7) = 182.34, p = 0.000); QB mean rank = 48.69; RB mean rank = 143.39; WR mean rank = 57.70; TE mean rank = 171.25; OL mean rank = 202.30; DL mean rank = 213.38; LB mean rank = 134.27; DB mean rank = 57.03. Specifically, QB, WR and DB experience significantly different BSE/T to the remaining five field positions (p = 0.000). Cumulative BSE/T for LB and RB was statistically different to BSE/T estimated for OL (p = 0.007; p = 0.041) and DL (p = 0.001; p = 0.004). Three unique cumulative BSE/T profiles were identified. QB, WR and DB (profile 1) experience, on average, fewer overall impacts, typically of high strain magnitude with longer time intervals (Fig. 3). BSE/T documented for LB and RB, (profile 2) consist of all levels of MPS magnitude of mid-range impact frequency and intervals. Finally, OL and DL (profile 3) positions experience the highest frequency of head impacts within short time intervals, predominately of low strain magnitude (Fig. 3). The TE position is unique in that the cumulative BSE/T was not significantly different to either field positions in profile 2 or profile 3, resulting in an overlap between the two profiles.

## Discussion

The complex nature of head injury and one’s history, predisposition, symptom expression, and RHI exposure results in an uncertainty on how to manage trauma to the brain. Focusing on one head impact metric to predict injury has proven challenging and typically results in low injury prediction sensitivity, particularly when using impact sensors to measure biomechanical metrics^43,48^. This research has employed a novel method of objectively quantifying RHI that captures a spectrum of severity in ASF. Difference in the characteristics of RHI between player field positions in National Football League was described.

This study yielded lower impact frequencies over a season in comparison to previous reports using impact sensors^36,38^, which include impacts sustained during practices. However, over the course of a 12-game season an average of 128 impacts per player was reported in collegiate football, comparable to the present findings^49^. Average impact frequency per game ranged from 2.3–22.1 depending on position, which are lower than previously reported excluding individual frequencies for RB, TE, and OL^36–38^. The higher frequencies documented for TE and RB can perhaps be attributed to their multiple roles on the field, and may also describe the lower frequencies reported for WR in comparison to Crisco and associates^37,38^. Their studies do not include frequency data for the TE position, and therefore may have been considered either a receiver or a lineman, thereby increasing the count for receivers. Most notable are the lower count estimations for the QB and DB. This discrepancy may be explained in a number of ways: as missed impacts from being limited to those within camera view, the tendency of impact sensors to over report hit counts, or differences in play between collegiate, high school, and professional level football. The OL and DL positions sustained the highest number of impacts throughout a game; predominantly from collisions to the top/front of the head (Fig. 1) which has been associated with detectable changes in the dorsolateral prefrontal cortex after sub-concussive head impacts^11^.

Based on the 32 game analysis, the average NFL football athlete may experience upwards of 1000 impacts from game play throughout their career, with closer to 6000 head impacts estimated for the greatest number of games played on record (Table 1). This estimation merely accounts for professional level game play and not the history of the athlete, where length of career and age of exposure influence risk^15,20–22^, and may, in addition to genetic and environmental factors, partly explain why not all former ASF athletes show signs of long-term clinical dysfunction^17,20,50^.

Reports identifying risk associated with RHI are limited in describing tissue deformation associated with common impacts experienced during a game^11,13,30,51^. Impact magnitudes obtained from the use of helmet impact sensors consistently report averages in the vicinity of 20 to 30 g and 1500 to 2000 rad/s^2^ for linear and rotational head accelerations, respectively^36,38,39,41,49,52^. These averages are found across all players, providing insufficient information on magnitude differences^36,39–41,52^. Further, peak head accelerations alone provide a less stable measure of impact severity than the brain tissue response^44,45^. Given that serum biomarker levels of axonal injury associate with higher hours of contact^8^, a better understanding of what magnitudes of tissue strain associate with game play provides a useful analysis for risk management strategies. Few differences in strain magnitudes were found between positions based on exemplar impacts in the current study, indicating that all positions may be exposed to a similar range in event severities. How the frequency of events distributed by strain magnitude provided more distinction between positions. QB, WR, and DB experienced the lowest impact frequencies but were among the positions most likely to sustain impacts of higher MPS magnitudes of above 26%, largely from head to ground contact (Fig. 1), and are among those reported as most vulnerable to sustaining ‘extreme’ hits^39^ and concussions^53^. Close to 95% of impacts received by OL, DL and TE were estimated at below 17% MPS and under 50% probability of concussion risk (Table 2; Fig. 2)^46,47^. Although these strain magnitudes are considered relatively low, repetitive low magnitude impact frequencies may exhibit structural and/or functional changes that are associated with long-term mental health concerns reported by current and retired football athletes^14,18,54–57^. Further, as little as 5–15% strain levels have been associated with functional impairment of signal transmission *in vitro*^58–62^.

Few impacts were recorded below 8% MPS across all positions. Pile-up situations presumably would consist of this level of impact although the nature of this scenario is such that impacts could not be confirmed and counted (Table 1). *In vitro* strains of 5% exhibit the minimum level of injury required to observe minor undulations and induce a calcium influx^61^. Observed undulations are caused by the immediate breakage and buckling of microtubules that are a consequence of mechanical failure^63,64^. Ahmadzadeh and researchers^65^ have also modeled macroscopic strains of 5% causing microscopic changes in the form of protein unfolding and microtubule rupture, a consistent neuropathology of tauopathy and brain diseases^20,66,67^. All impacts documented in this investigation were at or above these strain levels.

Time interval between impacts showed an inverse relationship with impact frequency (Fig. 3), which was an expected result. Impacts were documented on average every 6–30 minutes during game play in professional ASF. Oliver and researchers^8^ have demonstrated that elevations in biomarkers indicative of neuronal damage consistently increase at various time points throughout a season in ASF starter athletes (20–40 + plays/game), a population predominantly represented (starters) in the current study. Moreover, these measurements indicate that neurobiological recovery continues even with cessation of activity and RHI^6,8,14^. The relationship between interval and frequency becomes less predictable when examined individually at each magnitude level (Table 3). Although limited in sample size, the shortest time intervals between impacts of high strain magnitudes (>26% MPS) were reported for QB and TE. The importance of interval between high magnitude impacts has been described as an inconsistency between clinical symptom recuperation and neurobiological recovery^6,68^. Although time intervals are much longer in these reports, the very short intervals recorded here could influence the cumulative effect of sub-concussive impacts. Neurobiological damage may be present in the absence of clinical symptoms, and without a sufficient recovery there is a risk of cumulative injury^6,8^. The density to which sub-concussive impacts are experienced in succession may also collectively lead to concussion^35^.

The present study identified three profiles based on the combined characteristics of brain trauma exposure, BSE/T. Profile 1 identified positions that were exposed to low impact frequency of proportionately higher strain magnitudes above 17% MPS, experienced within 25–30 min time intervals. Player positions that are susceptible to a more even distribution of strain magnitudes at moderate frequencies within on average 13 min time intervals was identified as profile 2. Profile 3 was identified as positions that sustained the highest impact frequencies of magnitudes predominately below 17% MPS on average every 6–7 min time intervals. Positions that are recognized as more vulnerable to high magnitude events, such as DB, WR and QB, report the highest incidence of concussions^23^. Although lowest in frequency count, over 90% of the impacts documented for the QB were above moderate strain magnitudes, putting them above the 50% probability of concussion, essentially each time they are hit^46,47^. Sustaining a concussion does not inevitably lead to long-term mental disorders, nor does experiencing a greater number of concussive events consistently result in more severe cognitive decline among athletes^20^. This type of exposure, very few hits of high strains, may speak to the exhibition of immediate signs of acute injury, however coupled with sufficient recovery time could be less perilous for long-term damage^69^.

Long-term exposure to RHI associates with the inability to recover from injury, mental health disorders and, in most severe cases, degenerative brain disease years after exposure^14,20,70,71^. Consequently, positions identified in profile 3 are among the highest for confirmed cases of CTE^25^. Profile 2, perhaps the most dangerous, seems to have a less predictable brain trauma exposure, as they are exposed to relatively high impact frequency and are vulnerable to high impact magnitudes.

The TE position showed an overlap between the profile 2 and profile 3, possibly owing to their multiple roles on the field, and thereby successfully identifying TE as both a lineman and a short distance receiver. The RB position too, is unique in that they represent those players responsible for both running and passing plays, and is reflected in their exposure profile. With a low number of players on the field in a TE and/or RB position at any given time, they are considered among the riskiest positions for concussion and continuing neurological damage^23,25^. In light of the current distribution among position of various forms of diagnosed head injury, one can appreciate how an exposure profile may assist in the understanding of how tissue injury is manifested and expressed.

Few studies have examined RHI as a cumulative exposure metric based on multiple characteristics. These metrics are derived from self-reported history and/or head impact sensors, where typically higher exposure loads correlate with objective clinical measures^4,5,42,72^. The current study enhances our understanding of risks associated with RHI by providing tissue deformation magnitudes for a spectrum of common head impacts, giving contextual meaning to the cumulative strain experienced on the field. In practice, the RHI exposure profiles identified here can be used to estimate individualized exposures based on their position played, percentage of play, and number of games/seasons played. Exposure indices derived from this research can be used in comparative analyses involving current and former athletes, particularly useful in those with higher levels of play, presenting with neurological and cognitive health-related outcomes. As advancements in measurement techniques are becoming more refined and sensitive to all levels of head impacts, an objective method of measuring and quantifying RHI exposure is required to interpret clinical outcomes, guide legislation and intervention strategies and provide league officials, athletic directors, coaches and athletes information to make decisions concerning RHI in ASF.

Clinical outcomes, neither from individual events nor multiple events were documented in the current study, however, the range in impact frequency, strain magnitude and time between impacts demonstrates that RHI can be described using various combinations of exposure characteristics. There is agreement among scientists, neurologists and clinicians of the necessity to understand the relationship between the biomechanics of RHI, with neuroimaging, fluid biomarkers, genotype, symptom expression and altered behavior and cognition. One can appreciate that the population studied here may be at risk for a full spectrum of injury, regardless of field position, based on the intrinsic nature of the sport. The results, however, provide insight of the possible relationships between the characteristics of brain trauma exposure and specific outcomes. This measurement tool was effective at identifying the subtleties between player field positions and supports the notion that RHI can be described using various combinations of exposure characteristics. The brain’s many physiological responses under impact loading reflect the complexity of injury mechanisms^8,14,34,73,74^. Without an understanding of the event(s), there is little reference for change. Future applications using this method will be instrumental in connecting cumulative impact events to physiological response and injury outcomes. Connecting the characteristics of exposure to specific neuronal damage and neurobiological responses is required to estimate risk, establish dose-response relationships, and management of RHI. Establishing indices of exposure based on the interaction of impact frequency, strain magnitude and time interval will provide a measurement tool that, when applied universally, grants an opportunity to make informed decisions regarding risk mitigation strategies and public health policy for mental health protection and injury prevention for a myriad of sporting environments.

## Materials and Methods

### Game video analysis

Films of 32 professional ASF games from the 2009 to 2015 seasons were reviewed. One regular season game played by each of the 32 league teams was chosen at random for analysis. One starter player in each of eight positions was tracked and all head impacts were documented. When a tracked player was taken off the field, the substitution player was tracked for the same position. Each position was tracked throughout the entire game. Initial analysis involved Reviewer 1 clipping video of all head impacts that occurred and documenting the team name, position played, player name and number, type of event, time of impact, head location of impact, and estimated velocity level of impact based on recognized averages of jogging, running and sprinting speeds^75^. The impact confirmation process then consisted of every impact clip viewed by Reviewer 2, then Reviewer 3 to designate impacts as either confirmed or suspected (or multiple suspected), and discarding those that did not meet either definition. The final Reviewer 4 ensured that all documented confirmed impacts were consistent with the inclusion criteria. Velocity level was later confirmed using video analysis software (see Physical reconstruction of head impact events).

#### Head impact frequency counts

Impacts were designated as confirmed, suspected, or multiple. Impacts were logged as confirmed if: contact with the head was clearly visible; the event type could be determined; the head location could be determined. Suspected impacts were treated as events in which the player being tracked appeared to have experienced a head impact; however, due to any of the following reasons an impact could not be confirmed: poor video quality, poor camera angle of view, point of contact was obscured by another player. Multiple impacts were documented for circumstances in which players were tackling or being tackled and entered a pile-up situation, suggesting that multiple impacts were suspected; however, the specifics of those events could neither be distinguished nor confirmed.

#### Time interval between head impacts

Clock time of impact was recorded and used to calculate the time intervals. Total game time and time of play was used to calculate the percentage of stop time for all games independently. Interval between every impact was multiplied by the stop time specific to each game. Intervals were calculated in minutes. A two-minute interval was added between the last impact in the first quarter and the first impact in the second quarter, similarly between the third and fourth quarter. A 12-minute interval was added between impacts occurring on either side of half time. Two games went to overtime where a 5-minute interval was added between the end of the fourth and beginning of the overtime. Time intervals between each impact within level of magnitude were calculated. Impacts were further categorized into four time intervals for the calculation of BSE/T and are as follows; very low = <15 min, low = 15–30 min, moderate = 31–90 min, high = 91 min+.

### Physical reconstruction of head impact events

Four primary event types were reconstructed in laboratory; helmet, shoulder, hip/thigh and ground. All other events were documented as ‘other’. To appropriately reconstruct an event, the following five parameters were determined and categorized: velocity, location, orientation, effective mass, and compliance. These parameters create unique head kinematic motions that subsequently influence the magnitude of neural tissue loading and physical responses^46,47,76–85^. Inbound velocity, and head location and orientation were obtained during video analysis; effective mass and compliance were approximated using known measurements and available literature^86–88^.

#### Velocity, location, orientation

Inbound velocity was calculated by establishing the distance separating the player’s head from the contact surface (i.e. opponent shoulder) three to five frames prior to the impact using Kinovea software (version 0.8.20). Collision velocity (helmet, shoulder, hip/thigh) was calculated using field lines and video recording speed as described in a number of earlier studies^89,90^. Fall (ground) velocities were estimated by calculating the resultant velocity in a two-step process. Horizontal velocities were calculated using a marker system and perspective grid of known dimensions. The coordinates of the marker (representing camera movement) and the coordinates of the player’s head (representing camera + player movement) were documented for three frames prior to contact and velocity was calculated based on displacement measurements. The calculated velocity of the marker (camera) was subtracted from the player (camera + player) velocity for each frame and an average velocity was calculated. Vertical distances were established by measuring the distance from the player’s head to the ground two to three frames prior to contact using the known helmet width or length measurement as a reference^91^. All impacts were categorized into five velocity levels: Collisions; very low = <2.0 m/s, low = 2.1–4.5 m/s, moderate = 4.6–7.0 m/s, high = 7.1–9.5 m/s, very high = 9.6 + m/s, Falls; very low = <2.0 m/s, low = 2.1–4.0 m/s, moderate = 4.1–6.0 m/s, high = 6.1–8.0 m/s, very high = 8.1 + m/s.

**Figure 4:**
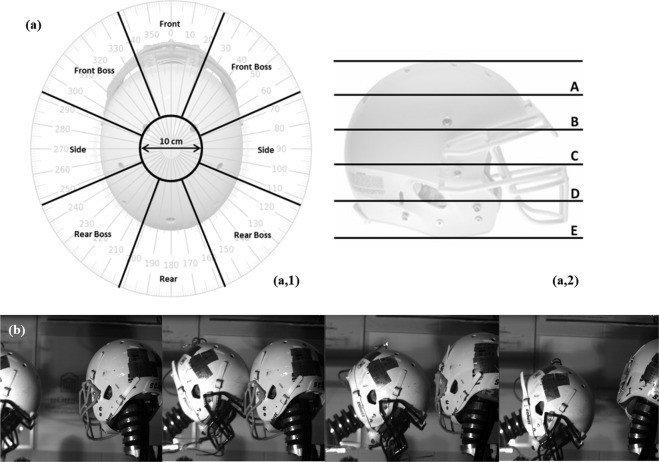
Location and direction estimation and validation for physical reconstruction of head impact events. (**a**) Top and side view of a football helmet demonstrating (1) the 8 sectors of 45° within the transverse plane, and (2) five levels of elevation used in the reconstruction protocol. (**b**) Validation of head motion post impact using high speed imaging.

Head locations were categorized as front, front boss, side, rear boss, rear and crown and were labelled using the reference system illustrated in Fig. 4. In the transverse plane, the head was divided into 8 sectors of 45°, representing 5 head locations, with eyes forward being 0°. All impacts occurring at the top of the helmet were categorized as crown (Fig. 4a-1). Side and boss locations were treated as one location independent of occurring on either the right or left side of the head. In the sagittal plane, the distance between the base and top of the helmet was divided into five equal levels of elevation (minus crown location) (Fig. 4a-2). Similar methods used and outlined by Rousseau^89^ deemed the level of precision was consistent with previous investigations. The orientation of impact was estimated within a tolerance of 15° as smaller increments have little effect on headform dynamic response^92,93^. Location and direction were verified using a high speed imaging PCI-512 Fastcam running at 2 kHz and Photron Motion Tools computer software (Photron, San Diego CA) (Fig. 4b).

#### Compliance, mass

**Figure 5:**
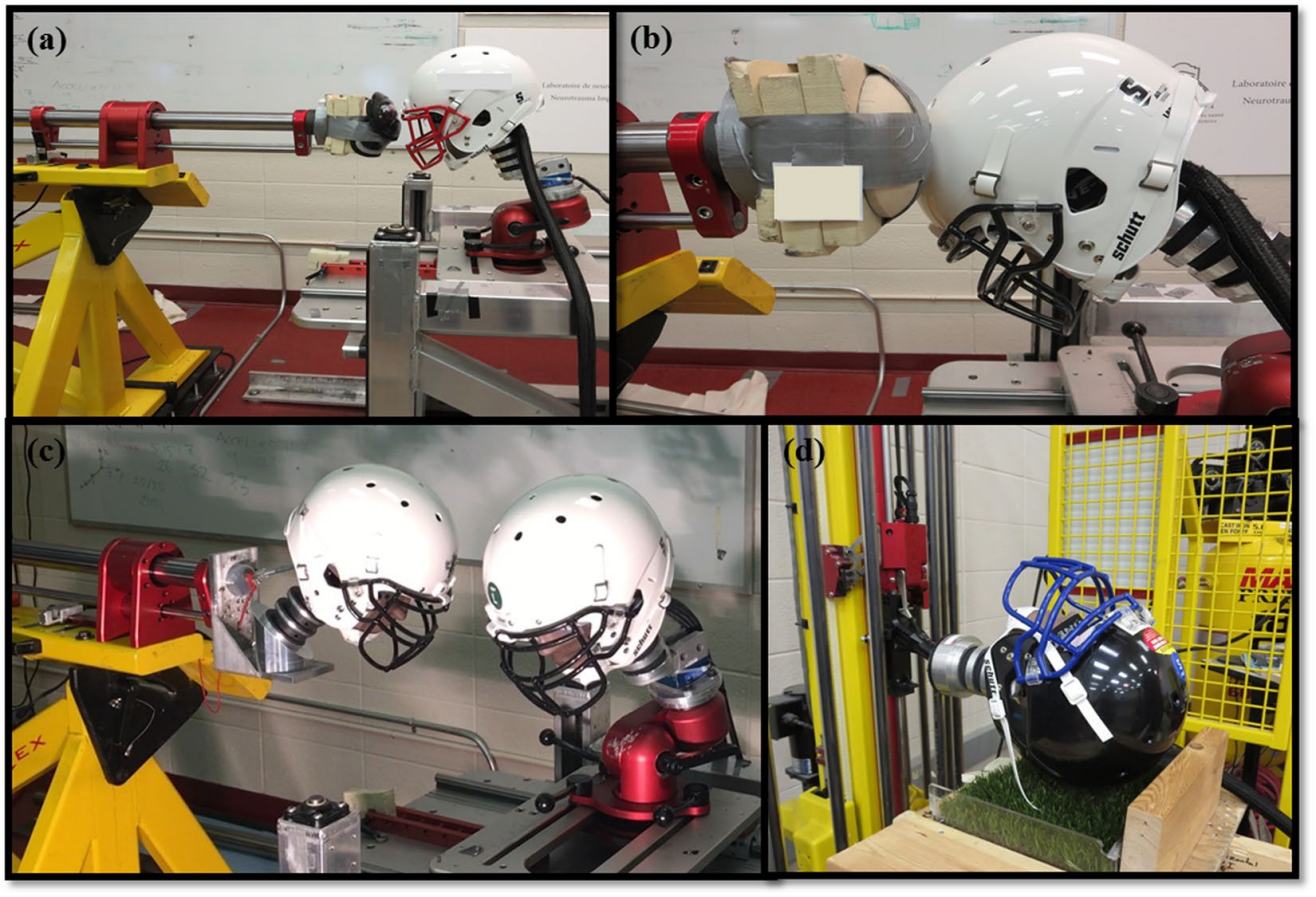
Equipment used for physical reconstructions of exemplar head impact events. (**a**) Shoulder events employing vinyl nitrile foam and shoulder pad cap attached to the arm of linear impactor system. (**b**) Foam impactor cap to replicate the compliance of hip/thigh events. (**c**) L-frame attachment for additional hybrid III head and neck for helmet impact events. (**d**) Hybrid III head attached to monorail drop system impacting field turf surface for ground events.

Compliance and mass were achieved using appropriate reconstruction equipment and selected based on the type of impact event. A pneumatic linear impactor (collisions) and a drop rig (falls) were used to replicate the four event types. The linear impactor consisted of a stationary steel frame secured to a cement floor supporting a 1.28 ± 0.01 m long cylindrical, free-moving impactor arm (13.1 ± 0.1 kg) (Fig. 5). A Hybrid III 50^th^-percentile adult male head form and neutral unbiased neck form^94^ (mass 6.65 kg ± 0.01 kg) was attached to a 12.78 ± 0.01 kg sliding table to allow for post impact movement. The inbound velocity matched the values obtained from the categorization of impacts. Shoulder and hip/thigh events utilized a nylon disc (diameter 13.2 mm) covered with a 142 mm thick layer of vinyl nitrile 602 foam attached to the impacting end of the arm to simulate human shoulder and hip compliance^88,89^ (Fig. 5a,b). Additionally, a pro football shoulder pad was attached to the impacting end for shoulder collisions (Fig. 5a). The striking mass was 15.5 kg and 15.2 kg for shoulder and hip/thigh events respectively, representing a realistic effective mass of player-to-player collisions^87,88^. Helmet events employed an additional Hybrid III head and neck form attached to the impacting end of the cylindrical arm via an L-frame and angled wedge to represent the striking player (Fig. 6c). Four different angled wedges (15°, 30°, 45° and 60°) were used to obtain the desired neck angle of the striking player. A heavier free-moving cylindrical impacting arm (16.1 ± 0.1 kg) and a stiff Hybrid III neck form were used for these collisions to represent the higher mass and minimal neck bending of a striking player^95^ for a total striking mass of 24.0 kg. A monorail drop tower with turf anvil was be used to simulate the ground events (Fig. 5d). The helmeted Hybrid III head and neutral unbiased neck form was attached to a guided rail system via a holding carriage. The assembly was dropped unrestrained from a predetermined height onto a turf surface to duplicate the contact surface of a football field.

An exemplar event was chosen for reconstruction for every permutation in impact condition (event type X velocity level X head location) experienced by each of the eight positions. This resulted in 249 head impact event exemplars. Table 4 presents the exemplar event type classification for each field position. All event reconstructions consisted of three impact trials for a total of 747 simulated impacts. Impacts that met the following criteria were chosen as exemplar events in this study: the event was captured on video, multiple lines were visible in a plane of view of the camera to determine plane perspective using distortion correction algorithms, and the head location could clearly be determined. If a condition consisted of two or three impacts than an average of the calculated velocities was used for the reconstruction. The midrange value of a velocity level (i.e. low collision = 3.25 m/s) was used for reconstructions in which the exemplar represented a category consisting of four or more impacts. Finally, for exemplars representing only one impact condition, the calculated velocity for that event was used for the physical reconstruction.

**Table 4:** Event type classification and exemplar totals for eight field positions.

	Event Type				Exemplar / Position
	Helmet *(n)*	Shoulder *(n)*	Hip *(n)*	Ground *(n)*	
QB	5	1		10	**16**
RB	14	13	6	16	**49**
WR	9	2	3	14	**28**
TE	11	11	3	12	**37**
OL	7	8	3	5	**23**
DL	11	9	3	8	**31**
LB	10	9	3	10	**32**
DB	5	9	4	15	**33**
**Total #**	**72**	**62**	**25**	**90**	

#### Collection system

Nine single-axis Endevco 7264C-2KTZ-2-300 accelerometers (Endevco, San Juan Capistrano CA) mounted in a 3-2-2-2 array in the headform were sampled at 20 kHz and filtered using the SAE J211 class 1000 protocol^96,97^. A SLICE free motion headform was outfitted with three single-axis accelerometers and 3 angular rate sensors fixed near the headform center of gravity. The accelerometer signals were passed through a TDAS Pro Lab system (DTS, Calabasas CA) before being processed by TDAS software. An electronic time gate measured the inbound velocity just prior to head impact using National Instrument’s VI Logger software.

### Finite element brain modelling

The University College Dublin Brain Trauma Model (UCDTBM) was used to calculate the brain deformations resulting from the exemplar head impacts. The UCDBTM model geometry was developed from CT and MRI imaging of the head of a male cadaver, and the version used in this research was composed of the scalp, skull, pia, falx, tentorium, CSF, grey and white matter, cerebellum, and the brain stem. Overall, the brain was composed of approximately 26,000 hexahedral elements^98,99^. Model material characteristics were established from earlier research^100–103^, presented in Table 5, modelled using a linearly viscoelastic model combined with large deformation theory. The behaviour of the tissue is characterized as viscoelastic in shear with a deviatoric stress rate dependent on the shear relaxation modulus, and defined as   elastic in compression. The interaction between the CSF and the brain represents the brain/skull interface and is modeled with solid elements with a high bulk modulus and low shear modulus to allow for sliding behavior similar to fluids. This interface uses a friction coefficient of 0.2 and the contact interaction specified no separation^104^. The shear modulus characteristics of the brain tissue, modeled as viscoelastic is given by Eq. (1):

1 $${\rm{G}}({\rm{t}})={{\rm{G}}}_{\infty }+({{\rm{G}}}_{0}-{{\rm{G}}}_{\infty }){e}^{-\beta t}$$

where G_∞_, is the long term shear modulus, G_0_, is the short term shear modulus, and *β* is the decay factor^99^.

**Table 5:** Brain tissue material properties and characteristics used for the UCDBTM components.

Material	Young’s modulus (MPa)		Poisson’s ratio	Density (kg/m^3^)
Scalp	16.7		0.42	1000
Cortical Bone	15000		0.22	2000
Trabecular Bone	1000		0.24	1300
Dura	31.5		0.45	1130
Pia	11.5		0.45	1130
Falx and Tentorium	31.5		0.45	1130
Brain	Hyperelastic		0.499981	1040
CSF	Water		0.5	1000
Facial Bone	5000		0.23	2100
	**Shear Modulus (kPa)**		**Decay Constant (s**^**−1**^**)**	**Bulk Modulus (GPa)**
	**G**_**0**_	**G**_**∞**_		
Cerebellum	10	2	80	2.19
Brain Stem	22.5	4.5	80	2.19
White Matter	12.5	2.5	80	2.19
Grey Matter	10	2	80	2.19

Validation of the model was conducted by comparing simulation responses to cadaveric testing that measured intracranial pressure^105^ and relative brain skull motion^106^. Comparisons to real world reconstructions of traumatic brain injury incidents were also conducted as a further validation^107^.

#### Magnitude of impact

Impact magnitude was measured and characterized using MPS. This metric has been identified as being among the best predictors of severity as it provides a more complete analysis of the brain’s mechanical response under loading that is difficult to capture using peak head acceleration alone^44,46,47^. MPS was calculated from exemplar head impact events using the three-dimensional head motion dynamic response curves obtained from the Hybrid III physical model reconstructions and applied to the centre of gravity of the finite element brain model^47^. Five MPS magnitude categories were established to capture a spectrum of severity that occurs in the sporting environment. These categories were based on biomechanical event reconstructive research and anatomical tissue analysis that associate with, physiological brain responses to axonal stretch, reported concussion, sub-concussion and clinical outcomes. Moderate magnitudes represent a category based on a 50% risk of concussion injury measured in the cerebrum white or grey matter^46,47,59^. Strains below this range were considered very low^60,61,65,108,109^ and low magnitudes^8,110^; high magnitudes correspond to those reported as average estimates to sustaining a concussive level injury^89,111^. A very high category was included to designate impacts that put athletes at substantially higher risk and represent a 50% risk of loss of consciousness and persistent symptoms^112,113^. MPS magnitudes for each condition were categorized as follows: very low = <8%, low = 8-16.9%, moderate = 17–25.9%, high = 26–34.9%, very high = 35%+. Strain magnitude values resulting from exemplar head impact reconstructions were assigned to each head impact within their represented impact condition category, thereby assigning an MPS magnitude to each head impact within the four primary event types.

### Cumulative RHI exposure

An exposure index, BSE/T, utilizing the three characteristics was created to quantify cumulative RHI for each field position throughout each game. This index was calculated by multiplying the frequency of impacts within each brain tissue strain magnitude category by its severity # (very low = 1; low = 2; moderate = 3; high = 4; very high = 5), then multiplied by the time interval in which those impacts were experienced (very low = 4; low = 3; moderate = 2; high = 1). This resulted in five values (one for each magnitude category) which were added together and divided by the total impact frequency experienced throughout the game to account for the number of impacts that created the RHI exposure.

### Statistical differences between American-style football player positions

Nonparametric rank based Kruskal-Wallis H tests were conducted to examine statistically significant effects of player field position on impact frequency, strain magnitude, time interval between impacts, and BSE/T. Impact frequency, strain magnitude, time interval and BSE/T variables were considered as dependent variables and eight player positions as independent variables. Frequencies, magnitudes, and intervals of confirmed head impacts resulting from the four primary event types were included in the analysis. Interval was included for games where >1 impact was documented within the same game: (QB, *n* = 24), (RB, *n* = 32), (WR, *n* = 26), (TE, *n* = 32), (OL, *n* = 32), (DL, *n* = 32), (LB, *n* = 31), (DB, *n* = 28). Post hoc Dunn adjusted by the Bonferroni correction for multiple tests were performed when significance was found. Additionally, a contingency table analysis was performed to examine the frequency distribution differences in strain magnitude categories amongst player field position. A Bonferroni correction was applied for multiple comparisons. Statistical level was set at α = 0.05 for all analysis and performed using IBM SPSS Statistics 24.0.

## Data Availability

Raw and processed physical and computational data, and game video clips are currently being stored at the University of Ottawa, Canada. It is available upon request.
